# Protein Kinase R in Bacterial Infections: Friend or Foe?

**DOI:** 10.3389/fimmu.2021.702142

**Published:** 2021-07-08

**Authors:** Robin Smyth, Jim Sun

**Affiliations:** ^1^ Department of Biochemistry, Microbiology and Immunology, University of Ottawa, Ottawa, ON, Canada; ^2^ Centre for Infection, Immunity and Inflammation, University of Ottawa, Ottawa, ON, Canada

**Keywords:** Protein Kinase R, bacterial infection, macrophage signaling, antibacterial defense, EIF2AK2, cell death, autophagy, inflammation

## Abstract

The global antimicrobial resistance crisis poses a significant threat to humankind in the coming decades. Challenges associated with the development of novel antibiotics underscore the urgent need to develop alternative treatment strategies to combat bacterial infections. Host-directed therapy is a promising new therapeutic strategy that aims to boost the host immune response to bacteria rather than target the pathogen itself, thereby circumventing the development of antibiotic resistance. However, host-directed therapy depends on the identification of druggable host targets or proteins with key functions in antibacterial defense. Protein Kinase R (PKR) is a well-characterized human kinase with established roles in cancer, metabolic disorders, neurodegeneration, and antiviral defense. However, its role in antibacterial defense has been surprisingly underappreciated. Although the canonical role of PKR is to inhibit protein translation during viral infection, this kinase senses and responds to multiple types of cellular stress by regulating cell-signaling pathways involved in inflammation, cell death, and autophagy – mechanisms that are all critical for a protective host response against bacterial pathogens. Indeed, there is accumulating evidence to demonstrate that PKR contributes significantly to the immune response to a variety of bacterial pathogens. Importantly, there are existing pharmacological modulators of PKR that are well-tolerated in animals, indicating that PKR is a feasible target for host-directed therapy. In this review, we provide an overview of immune cell functions regulated by PKR and summarize the current knowledge on the role and functions of PKR in bacterial infections. We also review the non-canonical activators of PKR and speculate on the potential mechanisms that trigger activation of PKR during bacterial infection. Finally, we provide an overview of existing pharmacological modulators of PKR that could be explored as novel treatment strategies for bacterial infections.

## Introduction

Interferon-induced, double-stranded RNA-activated protein kinase, also known as protein kinase R (PKR), is a ubiquitously and constitutively expressed serine-threonine kinase that is specifically found in vertebrate cells (1). PKR is encoded in humans by the *EIF2AK2* gene located on chromosome 2 and is 551 amino acids in length (2, 3). This kinase senses and responds to multiple types of cellular stress by regulating cell-signaling pathways involved in inflammation, cell death, and autophagy. As such, dysregulation of PKR expression or activation has been linked to multiple human diseases, including neurodegeneration, cancer, metabolic disorders, and viral infections [reviewed in-depth previously: (4–6)]. In particular, the most well-characterized function of PKR is to sense viral double-stranded RNA (dsRNA) for its canonical role in antiviral defense (7).

The best characterized transcriptional inducers of PKR are type I interferons (IFN). Type I IFN are produced in response to pathogen-associated molecular patterns (PAMPs) and signal through the IFNα/IFNβ receptor (IFNAR) to induce transcription of numerous genes that assist in antiviral defense [reviewed in-depth previously (8)]. These genes are referred to as interferon-stimulated genes (ISGs) and include *EIF2AK2*. Indeed, the PKR promoter contains an interferon-stimulated response element (ISRE), thus prompting transcription of the *EIF2AK2* gene in response to type I IFN signaling (9). The PKR promoter also contains a kinase conserved sequence upstream of the ISRE, which possesses binding sites for the transcription factors Sp1 and Sp3 (9, 10). Sp1 and Sp3 cooperatively activate basal PKR expression in the absence of IFN stimulation (10). The canonical activator of PKR is viral dsRNA (7); however, PKR can also be activated in response to a variety of stress signals, including serum starvation and endoplasmic reticulum (ER) stress (11). This activation of PKR in the absence of viral dsRNA is mediated by PKR protein activator (PACT). PACT is phosphorylated under cellular stress and physically interacts with PKR to trigger its activation (11). The more well-characterized roles of PKR include regulation of protein translation and apoptosis in response to viral infection, controlling cell proliferation and differentiation, and supressing tumour growth [reviewed in-depth previously: (4, 12)].

Structurally, PKR has a C-terminal kinase domain and an N-terminal dsRNA binding domain (dsRBD). The dsRBD consists of two dsRNA binding motifs (dsRBM1 and dsRBM2), both of which are required for the high-affinity interaction with viral dsRNA (13). Recognition and binding of dsRNA by the dsRBMs triggers homodimerization of PKR and its subsequent autophosphorylation (14, 15). PKR is autophosphorylated at multiple serine and threonine residues, including Thr446 and Thr451, which are consistently phosphorylated during PKR activation (14, 16). Once activated, PKR phosphorylates serine 51 (Ser51) on the alpha subunit of eukaryotic initiation factor-2 (EIF2α). PKR belongs to a family of four EIF2α kinases, all of which share the same substrate. The other three EIF2α kinases are heme-regulated inhibitor, PKR-like endoplasmic reticulum kinase (PERK), and general control non-depressible 2 (GCN2), which are activated by heme depletion (17), ER stress (18), and amino acid starvation (19), respectively.

Phosphorylation of EIF2α by PKR or any of the other three EIF2α kinases results in inhibition of protein translation. Mammalian EIF2 is critical for initiating polypeptide chain synthesis since it promotes the delivery of initiator methionyl transfer RNA (Met-tRNAi) to the 40S ribosome. EIF2α binds Met-tRNAi in a GTP-dependent manner, forming a ternary complex that interacts with the 40S subunit. Following Met-tRNAi delivery, EIF5 promotes GTP hydrolysis of EIF2-GTP, triggering the release of EIF2-GDP from the 48S initiation complex. EIF2-GDP must be regenerated to EIF2-GTP by the GTP exchange factor EIF2B, since EIF2-GDP is inactive. When Ser51 on EIF2α is phosphorylated, the affinity of EIF2 for EIF2B is increased up to 100-fold (20). Consequently, phosphorylated EIF2α competes with EIF2-GDP for binding of EIF2B. This competitive inhibition prevents the regeneration of active EIF2-GTP and as such, initiation of translation is substantially reduced (21). Functionally, this mechanism prevents the translation of both cellular and viral messenger RNA (mRNA), thereby inhibiting viral replication.

Although the canonical role of PKR is to inhibit protein translation during viral infection [reviewed in-depth previously: (12, 22–24)], PKR is in fact a versatile kinase that controls signal transduction pathways to mediate transcription and cellular processes. Given that PKR regulates critical immune cell functions in inflammation, cell death, and autophagy – processes that are critical for host immunity against bacterial infections – it is logical to expect that the role of PKR extends beyond that of antiviral defense. Surprisingly, the role of PKR in antibacterial defense is understudied and underappreciated relative to its role in antiviral defense. However, there is accumulating evidence demonstrating that PKR contributes significantly to the immune response to a variety of bacterial infections. This review provides an overview of immune cell functions regulated by PKR and includes an exhaustive summary of the current knowledge on the role and function of PKR in pathogenic bacterial infections. Specifically, we organized the sections by grouping bacteria under Gram-positive, Gram-negative, or mycobacteria, and included every report that we could find which linked PKR to pathogenic bacteria that cause human disease. We also review the non-canonical activators of PKR and speculate on the potential mechanisms that trigger PKR activation during bacterial infection. Finally, we provide an overview of existing pharmacological modulators of PKR that could be explored for treatment of bacterial infections.

## PKR in Immune Cell Function

### Inflammation

**Figure 1:**
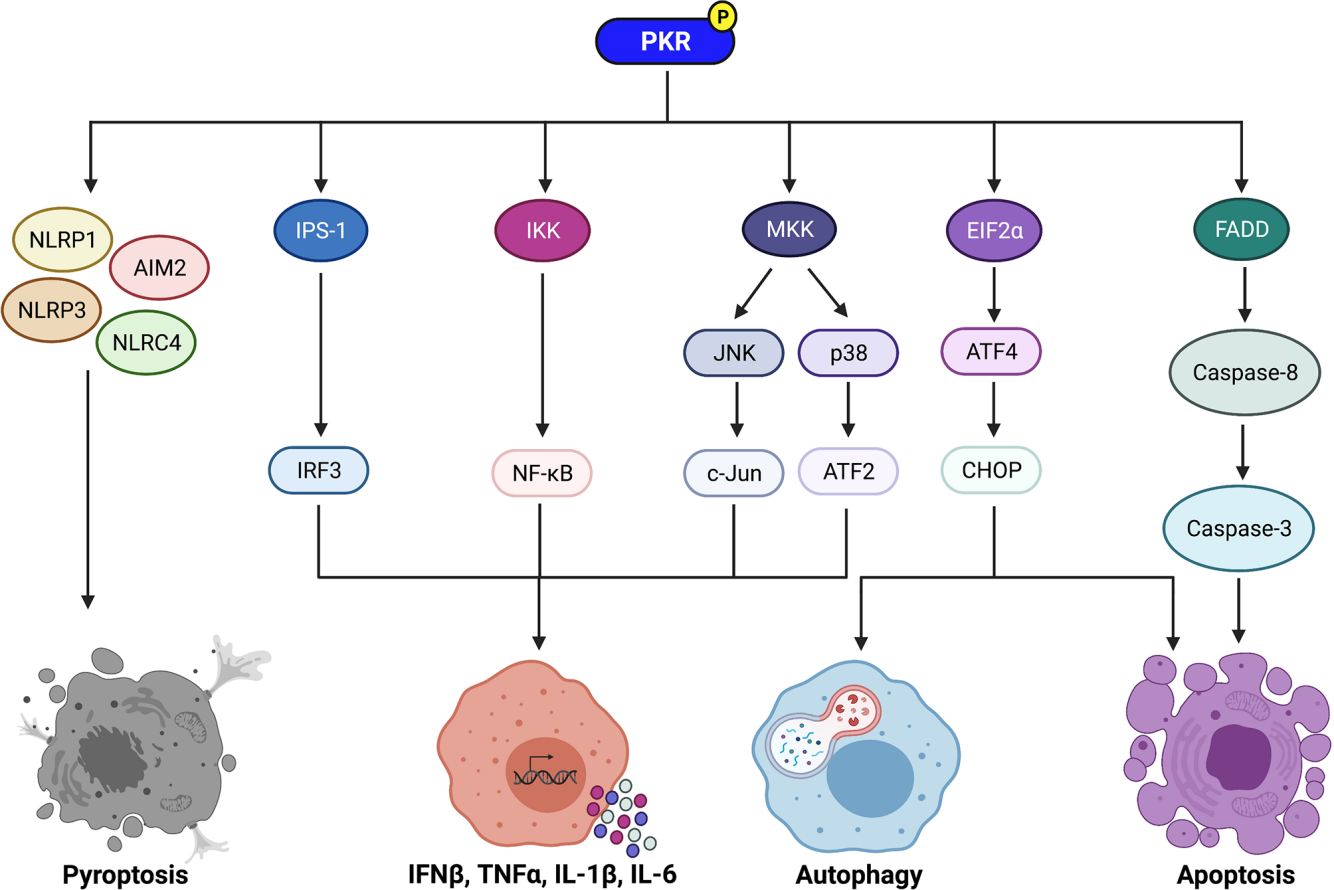
Signaling pathways regulated by PKR to control immune cell functions. PKR regulates downstream effectors such as IPS-1, IKK, and MKK to activate IRF3, NF-κB, and the MAP kinases JNK and p38, respectively. IRF3 induces transcription of IFNβ, whereas NF-κB induces transcription of pro-inflammatory cytokines such as TNFα, IL-1β, and IL-6. Active JNK and p38 trigger c-Jun and ATF2 activation, respectively, which also induce transcription of pro-inflammatory cytokines. PKR also plays a role in induction of pyroptosis via activation of the NLRP1, AIM2, NLRP3, and NLRC4 inflammasomes. Phosphorylation of EIF2α by PKR leads to increased translation of ATF4, which then increases expression of CHOP. ATF4 and CHOP trigger activation of autophagy by inducing transcription of essential autophagy genes. In addition, CHOP promotes apoptosis during periods of prolonged cellular stress. PKR can also induce apoptosis independently of EIF2α phosphorylation via activation of the FADD/caspase-8/caspase-3 pathway. Created with BioRender.com.

PKR regulates inflammation by activating multiple downstream effectors. One mechanism utilized by PKR to regulate inflammation is by activating mitogen-activated protein kinases (MAPK) such as p38 and c-Jun N-terminal kinase (JNK) (25–27) **(** **Figure 1** **)**. p38 and JNK trigger activating transcription factor-2 (ATF2) and c-Jun to induce the expression of proinflammatory cytokines such as interleukin (IL)-1β and tumour necrosis factor-alpha (TNFα) (28, 29) **(** **Figure 1** **)**. JNK is activated by MAPK kinase (MKK)4 or MKK7, whereas p38 is activated by MKK3 or MKK6. Depletion of PKR by stable knockdown impaired the phosphorylation of JNK and p38 in response to dsRNA or a mutant strain of vaccinia virus (25). Another group observed that PKR expression was required for full activation of JNK and p38 in response to polyinosinic:polycytidylic acid [poly(I:C)], lipopolysaccharide (LPS), IL-1β, and TNFα (26). In that same study, deletion of PKR in mouse embryonic fibroblasts (MEFs) was observed to inhibit MKK4 and MKK3/6 phosphorylation in response to the same stimuli. Interestingly, PKR deletion did not impact p38 or JNK activation in response to stressors that impact cellular components on a global scale, such as ultraviolet radiation, osmotic shock, and heat shock. The PKR-dependent stress stimuli were limited to pro-inflammatory ligands that bind distinct receptors, i.e. PKR as a receptor for dsRNA, CD14 and toll-like receptor (TLR)-4 for LPS, and the respective cytokine receptors for IL-1β and TNFα. Thus, it is suggested that PKR mediates activation of p38 and JNK in response to “receptor-mediated” pro-inflammatory stress stimuli, but not in response to “globally acting” stressors (26). Results from a different study support the observation that PKR activates p38 by acting upstream of MKK6 (27).

PKR can also regulate inflammation through its effects on NF-κB **(** **Figure 1** **)**. PKR indirectly activates NF-κB by activating IκB kinase (IKK) (30). Active IKK targets IκB, a negative regulator of NF-κB, for proteasomal degradation, thereby triggering its dissociation from NF-κB. NF-κB is then free to translocate to the nucleus, where it induces transcription of genes encoding pro-inflammatory cytokines such as IL-1, IL-6, and TNFα (30, 31) **(** **Figure 1** **)**. PKR triggers NF-κB activation in response to poly(I:C) and viral infection (30, 32, 33). While PKR physically associates with IKK (31), it remains unclear whether PKR is a structural or catalytic component in the activation of IKK (4, 12). Furthermore, although there are numerous reports of PKR activating NF-κB, there exists some contradictory evidence. Indeed, two independent studies have reported that PKR deficiency results in normal or only slightly decreased NF-κB activity in response to TNFα (34, 35). These findings suggest that PKR may play an important role in activating NF-κB in response to certain stimuli (e.g. dsRNA), but not others (e.g. TNFα treatment).

A third pathway by which PKR mediates inflammation is inducing type I IFN **(** **Figure 1** **)**. The canonical function of type I IFN is antiviral defense as they can directly limit intracellular viral replication and induce antiviral responses from T cells, natural killer cells, and B cells [reviewed in-depth previously: (36)]. However, there is increasing evidence to show that type I IFN also play a role in regulating inflammation, since they can alter the production of both pro- and anti-inflammatory mediators. For example, IFNβ treatment has been observed to increase MCP-1 and IP-10 production *via* STAT1 activation, which are crucial chemoattractants that recruit immune cells to the site of infection (37). On the other hand, IFNβ treatment also increases the production of the anti-inflammatory cytokine IL-10 and inhibits the secretion of pro-inflammatory cytokines such as IL-6 and TNFα *via* STAT3 activation in LPS-stimulated cells (37). In addition, type I IFN have been shown to regulate inflammation by controlling inflammasome activation (38). Interestingly, IFNβ was shown to enhance AIM2-dependent IL-1β secretion in response to *Francisella tularenis* or *Listeria monocytogenes* infection (39, 40) and mediates caspase-11-dependent pyroptosis during *Escherichia coli* and *Salmonella* Typhimurium infection (41, 42). These findings provide evidence that type I IFN regulate inflammation in the context of bacterial infections.

PKR activates IFNβ promoter stimulator-1 (IPS-1) signaling, which induces interferon regulatory factor 3 (IRF3) and subsequent transcription of IFNβ (43) **(** **Figure 1** **)**. Numerous studies have found that PKR deficiency impairs IFNβ production upon stimulation with poly(I:C) or viral infection (44). Curiously, Schultz and colleagues observed that IFNβ transcription was highly induced in PKR-deficient cells following encephalomyocarditis virus (EMCV) infection, but little or no IFNβ protein was produced (45). This suggested that PKR impacts the post-transcriptional regulation of IFNβ production. Indeed, further investigation revealed that IFNβ transcripts produced in EMCV-infected PKR-deficient cells completely lack a poly(A)tail (45). This indicates that PKR is required for the integrity of IFNβ mRNA and its translation into functional protein. As such, PKR has reported roles in increasing both transcription and translation of IFNβ. Since type I IFN modulates inflammation, the ability of PKR to induce type I IFN is another mechanism that allows the kinase to regulate the inflammatory response.

Collectively, PKR plays a central role in regulating inflammation through numerous downstream effectors, including MAPK p38 and JNK, NF-κB, and type I IFN **(** **Figure 1** **)**. Although acute inflammation assists with microbial clearance, chronic inflammation can result in tissue damage and deleterious effects to the host. In addition, many of the signaling pathways that regulate inflammation also have key roles in the control of cell death, a major cellular response that has consequences for bacterial infections. Inflammation must therefore be tightly regulated to achieve an optimal outcome for the host during bacterial infection.

### Cell Death

As mentioned in the previous section, inflammation and cell death share common signaling pathways and are tightly intertwined. It is thus unsurprising that PKR has reported roles in regulating cell death pathways such as apoptosis and pyroptosis. PKR was first shown to induce apoptosis in 1994, when expression of the kinase in HeLa cells triggered rapid apoptosis, an effect that was not observed in cells expressing a mutant form of PKR (46). Later studies using MEFs from PKR knockout mice or NIH3T3 cells expressing a catalytically inactive PKR mutant reinforced the finding that PKR plays a pro-apoptotic role during cellular stress (47, 48). The ability to induce apoptosis of virus-infected cells is now a well-known function of PKR (4, 49). Indeed, PKR regulates apoptosis in response to numerous viruses, including poxviruses, influenza, and EMCV (50–52). Importantly, PKR can induce apoptosis in the absence of viral infection, such as in response to LPS, TNFα, serum starvation, or ER stress (47, 48, 53, 54), suggesting a role for this kinase in non-viral contexts. PKR-dependent apoptosis in the absence of viral infection is reported to rely on PACT, which triggers PKR activation in response to numerous stressors such as serum withdrawal and ER stress (11, 55).

PKR regulates apoptosis through EIF2α-dependent mechanisms (32, 47) **(** **Figure 1** **)**. Phosphorylation of EIF2α results in repression of global protein translation but preferential translation of activating transcription factor-4 (*ATF4*) mRNA (56). ATF4 increases expression of C/EBP homologous protein (CHOP), a transcription factor that promotes apoptosis during periods of prolonged cellular stress. CHOP induces apoptosis by increasing expression of the pro-apoptotic protein Bim and decreasing expression of the anti-apoptotic protein Bcl-2 (57, 58). PKR can also induce apoptosis independently of EIF2α phosphorylation. One such mechanism is through activation of the FADD/caspase-8/caspase-3 pathway (59–61) **(** **Figure 1** **)**. In addition, NF-κB, ATF-3, and p53 are downstream effectors of PKR that are speculated to contribute to PKR-mediated apoptosis (4, 12), although the exact mechanisms remain unclear.

In addition to apoptosis, PKR is reported to regulate inflammasome activation and pyroptosis **(** **Figure 1** **)**. Lu and colleagues observed that activation of PKR was triggered by multiple inflammasome activators, and deletion of PKR inhibited high mobility group protein B1 (HMGB1) release, IL-1β secretion, and caspase-1 activation in response to inflammasome-inducing stimuli (62). Importantly, deletion of PKR also prevented cell death of macrophages treated with inflammasome activators. In the same study, PKR was shown to physically associate with NLRP1, NLRP3, NLRC4, and AIM2 inflammasomes. A non-phosphorylatable PKR mutant failed to bind NLRP3 and was unable to activate the inflammasome, indicating that phosphorylated PKR physically interacts with inflammasomes to induce their activation (62).

However, the role of PKR in pyroptosis remains controversial, as a study by Yim et al. reported that PKR represses inflammasome activation (63). Nigericin-treated peritoneal macrophages from PKR knockout or kinase-dead PKR mice resulted in elevated levels of IL-1β and IL-18, and enhanced caspase-1 activity. Ablation of PKR expression or kinase activity also promoted the expression or assembly of inflammasome components such as NLRP3 and pro-IL-1β. Since EIF2α phosphorylation impairs protein translation, the authors speculated that PKR represses translation of inflammasome constituents by its kinase activity on EIF2α. Indeed, pre-treatment of peritoneal macrophages with the small molecule ISRIB, which counteracts the effect of EIF2α phosphorylation, increased the expression of pro-IL-1β. Yim and colleagues attribute the discrepancy between their findings and those from Lu et al. to a difference in the mouse models used (63). Notably, Lu et al. used mice from a mixed 129Sv/BALB/c background, which have attenuated inflammasome activity due to diminished caspase-11 expression (64). To complicate matters further, He et al. showed that PKR is dispensable for inflammasome activity (65). However, the *in vitro* differentiation of mouse macrophages from this study is considered to be the source of the discrepancy compared to findings from the Yim et al. study, where primary macrophages were used without further manipulation *in vitro* (63). Altogether, these conflicting reports show that the animal and cellular model is a key determinant in whether PKR mediates inflammasome activation and pyroptotic cell death.

Nevertheless, the role of PKR in cell death has major implications for bacterial infections because the mode of cell death in bacteria-infected cells influences the outcome of infection. For example, apoptotic cell death is generally considered as a pro-host response during *Mycobacterium tuberculosis* infection, since it enhances cross-priming of T cells and limits inflammation (66). In contrast, pyroptotic cell death is considered as an anti-host response during *M. tuberculosis* infection, since it results in bacterial dissemination and host tissue damage (67, 68). As such, the ability of PKR to regulate cell death pathways such as apoptosis and pyroptosis is pertinent for host immunity against bacterial pathogens.

### Autophagy

PKR has been shown to induce autophagy, which may be a mechanism to balance its role in activating cell death and inflammation. Autophagy is a homeostatic process that generates nutrients by degrading cytoplasmic constituents, and this pathway is speculated to be protective against cell death (69). Although the canonical targets of autophagic degradation are organelles and proteins, it is now known that the autophagy pathway can selectively target pathogens for degradation in a process termed xenophagy (70, 71). Indeed, the autophagy pathway has been shown to degrade intracellular bacteria, viruses, and parasites (70–73). In the context of bacterial infections, selective autophagy allows for progressive elimination of bacteria (73), decreased bacterial burden (71), and improved control of inflammation (74). As such, autophagy is a critical pathway in antibacterial defense.

There is increasing evidence for PKR’s role in autophagy. An initial study by Tallóczy and colleagues found that EIF2α phosphorylation by the yeast EIF2α kinase GCN2 was essential for starvation-induced autophagy of yeast cells (75). Expression of PKR in GCN2-disrupted yeast rescued autophagy in these cells, indicating a role for PKR in induction of autophagy. A follow-up study by the same group showed that PKR can induce autophagy during viral infection (76). Infection of PKR knockout MEFs with a Herpes Simplex Virus-1 (HSV-1) mutant lacking the PKR-inhibiting virulence factor ICP34.5 significantly inhibited colocalization of virions with autophagosomes and resulted in increased viral titres compared to wild-type cells (76). More recently, Ogolla and colleagues reported that PKR induces autophagy in RAW264.7 macrophages during infection with the parasite *Toxoplasma gondii* (77). Indeed, PKR expression was required for LC3 accumulation around the parasite and lysosomal fusion with vacuole-containing *T. gondii* in macrophages, which are crucial events during selective autophagy. The autophagy-inducing role of PKR during *T. gondii* appears to be critical for controlling infection, as PKR knockout mice exhibited higher parasite loads compared to wild-type mice (77).

The mechanism of PKR-dependent induction of autophagy is likely through phosphorylation of EIF2α **(** **Figure 1** **)**. Indeed, Tallóczy and colleagues observed that MEFs expressing a non-phosphorylatable EIF2α mutant displayed diminished xenophagic degradation of HSV-1 proteins to the same extent as PKR knockout MEFs, and the viral titres were equivalent between the two cell-lines (76). This indicates that PKR-induced autophagic degradation of HSV-1 is mediated through phosphorylation of EIF2α. Phosphorylation of EIF2α increases the expression of transcription factors ATF4 and CHOP, which then induce transcription of essential autophagy genes such as *Map1lc3b*, *Atg12*, *Atg3*, *Atg7*, and *Becn1* (78). It is also possible that PKR induces autophagy through its downstream effects on MAPK p38 and JNK. Although the specific mechanisms by which p38 and JNK activate autophagy during bacterial infection have not yet been elucidated, these MAPK are reported to induce autophagy during starvation through indirect effects on Beclin-1, a crucial protein in the autophagy pathway. Specifically, p38 activates MK2 and MK3, which results in the phosphorylation of Beclin-1 (79). In contrast, JNK phosphorylates a negative regulator of Beclin-1, Bcl-2 (80), to trigger its dissociation from Beclin-1 (81). Given the critical role of autophagy against intracellular bacterial pathogens, the ability of PKR to trigger autophagy is likely a key cellular response to certain bacterial pathogens.

## PKR in Gram-Positive Bacterial Infections

### Staphylococcus aureus

*Staphylococcus aureus* is a Gram-positive, facultative intracellular bacterium that is commonly found in the upper respiratory tract and skin flora of humans. Although *S. aureus* is typically a commensal bacterium, it can become an opportunistic pathogen and cause a range of illnesses with varying severity, including cellulitis, osteomyelitis, pneumonia, and meningitis. *S. aureus* was initially characterized as an extracellular bacterium. However, it is now understood that *S. aureus* is phagocytosed by neutrophils and macrophages, where it manipulates the phagosome maturation pathway to avoid lysosomal degradation (82, 83). *S. aureus* secretes a pore-forming toxin known as α-toxin, which assists *S. aureus* in escaping from macrophage phagosomes (82) and leads to activation of the autophagy pathway (84). PKR was first suspected to play a role during *S. aureus* infection when Kloft and colleagues observed that autophagy is activated in α-toxin-treated HaCaT cells and that phosphorylation of EIF2α was required for the accumulation of autophagosomes (85). Further examination revealed that both PKR and GCN2 are phosphorylated in response to α-toxin, whereas PERK is not. These findings suggest that PKR- and/or GCN2-mediated phosphorylation of EIF2α activates autophagy during *S. aureus* infection (85). However, further investigation is required to determine which of these EIF2α kinases is responsible for this effect. Interestingly, PKR might also play a proapoptotic role during *S. aureus* infection, although the current evidence is limited. Treatment of cardiac cells with RNA extracted from *S. aureus* was shown to trigger PKR activation and induce apoptosis, whereas cells treated with a PKR inhibitor were resistant to apoptosis (86) **(** **Table 1** **)**. *S. aureus* RNA induced cleavage of capase-8, caspase-3, and caspase-9, an effect that was prevented following treatment with a PKR inhibitor (86). As such, PKR may activate caspase-8- and caspase-9-mediated apoptosis during *S. aureus* infection. Although the findings from the aforementioned studies suggest a role for PKR in the antibacterial response to *S. aureus*, neither of these studies examined the overall effect of PKR expression on the control of bacterial replication. Furthermore, these studies did not use live *S. aureus* infection, but instead examined the effect of bacterial RNA or α-toxin. As such, future investigation is required to determine whether PKR plays a role during infection with the live bacterium.

**Table 1:** Role of PKR in different bacterial infections.

Bacterium	Experimental model	Method of PKR modulation	Live bacterium	Effect of PKR modulation^a^	Bacterial burden^a^	Citation
***S. aureus***	Human cardiac myocytes	Pharmacological inhibition	No (RNA)	Decreased apoptosis	N.D	(86)
***B. anthracis***	Mouse BMDMs	Genetic deletion	Yes	Decreased apoptosis	N.D	(87)
	Mouse peritoneal macrophages	Genetic deletion	No (toxin)	Decreased pyroptosis	N.D	(62)
				Decreased inflammasome activation		
	J774 macrophages	siRNA knockdown	No (toxin)	Decreased pyroptosis	N.D	(88)
				Decreased inflammasome activation		
***L. monocytogenes***	Mouse BMDCs and BMDMs	Pharmacological inhibition	Yes	Reduced expression of CHOP	N.D	(89)
***S.* Typhimurium**	Mouse BMDMs	Genetic deletion	Yes	Decreased apoptosis	N.D	(87)
	Mouse peritoneal macrophages	Genetic deletion	Yes	Decreased pyroptosis	N.D	(62)
				Decreased inflammasome activation		
***E. coli***	C57BL/6J mice	Genetic deletion	Yes	Decreased inflammasome activation	Decreased (spleen, peritoneal cavity)	(62)
	Mouse peritoneal macrophages		Yes	Decreased pyroptosis	N.D	
				Decreased inflammasome activation		
	Mouse BMDCs		No (RNA)	Decreased inflammasome activation		
	C57BL/6J mice	Genetic deletion	Yes	N.D	Unaffected (lungs, liver, blood, spleen)	(90)
	Mouse peritoneal macrophages			Decreased IFNα and IFNβ	N.D	
	C57BL/6J mice	Genetic deletion	Yes	Decreased IL-1β mRNA in the liver	Unaffected (blood)	(91)
	Human cardiac myocytes	Pharmacological inhibition	No (RNA)	Decreased apoptosis	N.D	(86)
***Y. pseudotuberculosis***	Mouse BMDMs	Genetic deletion	Yes	Decreased apoptosis	N.D	(87)
***C. trachomatis***	MEFs	Genetic deletion	Yes	Increased bacterial invasion	N.D	(92)
	Human mDCs	Pharmacological inhibition	Yes	Decreased IFNβ	N.D	(93)
	Mouse BMDMs	Genetic deletion		Decreased IFNβ mRNA		
***L. pneumophilia***	U937 macrophages	shRNA knockdown	Yes	Decreased IL-6	Unaffected	(94)
***M. bovis* BCG**	Human primary monocytes	Pharmacological inhibition	Yes	Decreased IL-6, TNFα, IL-10	N.D	(95)
***M. tuberculosis***	C57BL/6J mice	Genetic deletion	Yes	No effect	Unaffected (spleen, lungs)	(96)
	THP-1 monocytes	Genetic deletion	Yes	N.D	Increased	(97)
		Pharmacological activation			Decreased	
	THP-1 macrophages	Genetic deletion	Yes	Decreased selective autophagy	Increased	(98)
		Genetic overexpression		Increased selective autophagy	Decreased	

^a^Based on “Method of PKR modulation” column. N.D, not determined.

### Bacillus anthracis

*Bacillus anthracis* is a Gram-positive, extracellular bacterium that is the causative agent of anthrax. *B. anthracis* secretes a major virulence factor known as lethal toxin, which enters host cells and cleaves MAPK kinases to impair MAPK signaling pathways (99). In doing so, lethal toxin disrupts crucial processes such as proliferation, survival, and inflammation in host cells. Anthrax lethal toxin has been shown to trigger phosphorylation of PKR in murine peritoneal macrophages (62), which suggests that PKR would be activated by *B. anthracis* infection. The role of PKR during *B. anthracis* infection remains unclear; however, there is evidence to suggest that PKR regulates cell death during infection with this bacterium. Indeed, Hsu and colleagues observed that deletion of PKR in bone marrow-derived macrophages (BMDMs) infected with live *B. anthracis* had markedly reduced apoptosis levels compared to wild-type macrophages (87) **(** **Table 1** **)**. Further examination into the mechanism revealed that PKR is required for TLR4-dependent apoptosis of *B. anthracis*-infected macrophages (87). Hett and colleagues provided further evidence that PKR regulates TLR4-dependent apoptosis in response to *B. anthracis*, since pharmacological inhibition of PKR protected LPS-sensitized macrophages from apoptosis in response to lethal toxin (88). Interestingly, Lu and colleagues observed that PKR was required for inflammasome activation in lethal toxin-treated mouse peritoneal macrophages, as indicated by impaired caspase-1 activation, IL-1β cleavage, and HMGB1 release in PKR-deficient cells (62) **(** **Table 1** **)**. Furthermore, PKR deficiency protected macrophages from lethal toxin-induced cytotoxicity. Consistent with the findings from Lu et al., Hett and colleagues reported that PKR is required for pyroptosis in response to lethal toxin challenge (88). Indeed, PKR knockdown protected J774 macrophages from cell death following treatment with lethal toxin, and was accompanied by reduced caspase-1 activity and IL-18 secretion **(** **Table 1** **)**. Interestingly, lethal toxin was not observed to induce PKR phosphorylation, and treatment with pharmacological inhibitors of PKR did not protect macrophages from lethal toxin-induced cell death. These findings indicate that the catalytic activity of PKR is not required for PKR-dependent pyroptosis in response to lethal toxin. As such, the authors speculate that PKR mediates activation of pyroptosis through physical interactions with inflammasomes (88). Since PKR regulates inflammasome activation in response to anthrax lethal toxin, it is possible that PKR would have the same activity in response to infection with live *B. anthracis*. However, findings from studies focusing on one virulence factor *in vitro* at the expense of studying whole organism infections with the live bacterium must be interpreted with caution. For example, Kang and colleagues reported that *B. anthracis* spores and lethal toxin induce IL-1β *via* functionally distinct pathways, demonstrating that different components of the same bacterium can mediate different signaling pathways (100). In fact, spore-induced IL-1β was observed to limit *B. anthracis i*nfection, whereas lethal toxin-induced IL-1β enabled *B. anthracis* to escape host defenses (100). Furthermore, although PKR expression was reported by Hsu et al. to be required for TLR4-dependent macrophage apoptosis in response to live *B. anthracis* infection, Moayeri and colleagues reported later that same year that lethal toxin-mediated lethality in mice was independent of TLR4 function (101). Altogether, these studies emphasize the need for *in vivo* studies with the live bacterium, since experiments focusing on single components/virulence factors of a bacterium *in vitro* can produce different results. In addition, the overall effect of PKR expression on bacterial burden during live *B. anthracis* infection has not been investigated.

### Listeria monocytogenes

*Listeria monocytogenes* is a Gram-positive, facultative intracellular bacterium that causes listeriosis, which can manifest as sepsis, meningitis, pneumonia, urinary tract infections, and gastroenteritis (102). This bacterium has been shown to induce phosphorylation of EIF2α in the murine macrophage cell-line RAW264.7, which indicates that PKR or another EIF2α kinase is activated during *Listeria* infection (103). Expression of a non-phosphorylatable mutant of EIF2α in MEFs resulted in increased bacterial invasion, suggesting an important role for EIF2α kinases in the antibacterial response to *Listeria* (103). Indeed, our group observed that *L. monocytogenes* infection triggers increased levels of total and phosphorylated PKR protein in the human macrophage cell-line THP-1 (98). Since *L. monocytogenes* is established to invade the cytosol and initiate a type I IFN response, and type I IFN signaling induces PKR transcription, Valderrama and colleagues examined the effect of *L. monocytogenes* infection on *EIF2AK2 (PKR)* mRNA levels (89). As expected, *L. monocytogenes* infection resulted in increased PKR transcription levels in murine bone marrow-derived dendritic cells (BMDCs) and BMDMs. Interestingly, an LLO knockout strain of *L. monocytogenes*, which lacks the LLO virulence factor required for phagosome escape, was able to induce transcription of PKR, although not to the same extent as wild-type *L. monocytogenes* (89). This indicates that cytosolic localization of *L. monocytogenes* is not required to induce transcription of PKR. Further investigation revealed that murine myeloid cells treated with a PKR inhibitor have reduced expression of CHOP following *L*. *monocytogenes* infection (89) **(** **Table 1** **)**. CHOP expression is increased following EIF2α phosphorylation and induces a number of effects such as proinflammatory cytokine secretion and inflammasome activation (104–107). As such, the authors speculate that PKR-dependent activation of CHOP triggers an inflammatory response to *L. monocytogenes* infection. However, this inflammatory role of PKR during *Listeria* infection may be harmful to the host, since CHOP knockout mice had decreased splenic cell death, decreased bacterial proliferation, and better survival compared to wild-type mice (89). Nevertheless, the direct effects of PKR modulation on *L. monocytogenes* survival and host outcome have yet to be studied.

## PKR in Gram-Negative Bacterial Infections

### Salmonella enterica

*Salmonella enterica* serovar Typhimurium is a Gram-negative, facultative intracellular bacterium that causes gastroenteritis in humans. *Salmonella* Typhimurium can infect both humans and animals, and infection is commonly acquired by consuming contaminated food products. *S.* Typhimurium infection has been observed to increase mRNA levels of *EIF2AK2* in MEFs (108), and we and others have reported that *S.* Typhimurium increases total and phosphorylated PKR protein levels in macrophages (87, 98). These findings suggest that PKR plays a role in the antibacterial response to *Salmonella*. Indeed, PKR has been shown to regulate host cell death during *Salmonella* infection. For example, Hsu et al. reported that PKR knockout BMDMs are resistant to apoptosis induced by *S*. Typhimurium infection in comparison to wild-type macrophages (87) **(** **Table 1** **)**. Macrophages expressing a non-phosphorylatable mutant of EIF2α were also less susceptible to *Salmonella*-induced apoptosis, although a residual apoptotic response in these macrophages suggested the existence of another PKR-dependent pro-apoptotic pathway. The authors examined the levels of *Salmonella*-induced apoptosis in IRF3 knockout macrophages, and these macrophages were also resistant to *Salmonella*-induced apoptosis (87). As such, PKR may regulate two pro-apoptotic pathways during *Salmonella* infection, one involving EIF2α and the other involving IRF3. Interestingly, the apoptotic response to *Salmonella* was considerably reduced in BMDMs from TLR4-deficient mice, indicating that PKR is required for TLR4-dependent apoptosis during *Salmonella* infection (87). PKR is also reported to play a role in pyroptosis activation during *Salmonella* infection. Lu and colleagues observed that PKR deficiency in *S.* Typhimurium-infected murine peritoneal macrophages significantly inhibited caspase-1 activation, IL-1β cleavage, and HMGB1 secretion, as well as *Salmonella*-induced cell death (62) **(** **Table 1** **)**. PKR was shown to physically associate with inflammasomes in response to a variety of pyroptosis-inducing stimuli, and PKR expression was required for inflammasome activation. However, the specific mechanism of inflammasome activation by PKR remains unknown. Taken together, the findings from Hsu et al. and Lu et al. indicate that PKR is an important mediator of both apoptosis and pyroptosis in *Salmonella-*infected macrophages. However, the overall effect of PKR on bacterial survival remains to be elucidated.

Lastly, findings from Yeung and colleagues loosely suggest a role for PKR in the proper functioning of *Salmonella-*infected macrophages (109). The outcome of *Salmonella* infection is largely influenced by how the bacteria initially interact with macrophages, however the human macrophage factors required for *Salmonella* uptake are incompletely understood. A genome-scale CRISPR knockout library screening of THP-1 macrophages to identify loss-of-function mutations conferring resistance to *Salmonella* uptake identified NHLRC2, a gene involved in actin dynamics (109). NHLRC2 mutant macrophages were hyperinflammatory, unable to interact and phagocytose *S.* Typhimurium, and exhibited atypical morphology. Interestingly, PKR was shown to physically associate with NHLRC2, and NHLRC2 knockout macrophages had reduced PKR expression (109). Since PKR expression appears to be linked to NHLRC2 expression, and these two proteins physically interact, perhaps PKR contributes to NHLRC2-dependent uptake of *Salmonella* and the proper functioning of infected macrophages.

### Escherichia coli

*Escherichia coli* is a Gram-negative bacterium that can either be harmless or pathogenic depending on the particular strain. Some strains of *E. coli* are part of the normal intestinal microbiota, whereas other strains can cause diarrhea, urinary tract infections, or other illnesses. There have been a few studies examining the effect of PKR deletion during *E. coli* infection both *in vitro* and in the mouse model. Lu et al. reported that PKR expression and phosphorylation is triggered in murine peritoneal macrophages following *E. coli* infection *in vitro* (62). It was observed that *E. coli*-induced pyroptosis was severely impaired in infected PKR knockout macrophages, as indicated by decreased cell death and impaired IL-1β production **(** **Table 1** **)**. Furthermore, transfection with *E. coli* RNA in BMDCs significantly activated capase-1 and stimulated IL-1β cleavage in wild-type cells but not PKR knockout cells (62) **(** **Table 1** **)**. Lu and colleagues also performed *in vivo* experiments and observed that serum IL-1β, IL-18, and HMGB1 levels were significantly reduced in *E. coli*-infected PKR knockout mice compared to control mice. Notably, PKR knockout mice had significantly lower titers of *E. coli* in the spleen and peritoneal cavity compared to control mice (62) **(** **Table 1** **)**. Altogether, the findings from this study suggest that PKR plays a role in inflammasome activation during *E. coli* infection, and PKR expression appears to be conducive for *E. coli* persistence. However, it is difficult to interpret the results from these *in vivo* studies since non-virulent *E. coli* was used, as reflected by >10^9^ CFU challenge doses (62). In contrast, a recent study from the same group using virulent *E. coli* showed that genetic deficiency or pharmacological inhibition of PKR did not affect bacterial loads in *E. coli*-infected mice (90) **(** **Table 1** **)**. The discrepancy in findings between these two studies was suggested by the authors to be due to differences in the route of *E. coli* infection (90). In the first report, Lu et al. performed intraperitoneal infection, whereas the second study used intravenous infection (62, 90). Overall, more careful *in vivo* studies using virulent *E. coli* (typically reflected by dose challenges of 10^7^ CFU or lower) must be performed before conclusions can be drawn.

A role for PKR in inflammasome activation was also reported by Poon and colleagues (91). This group observed that PKR knockout mice had diminished peripheral inflammatory responses following subcutaneous *E. coli* challenge. Indeed, PKR deficiency resulted in decreased IL-1β mRNA expression in the livers of infected mice (91) **(** **Table 1** **)**. However, PKR deletion had no effect on the bacterial burden of *E. coli*-infected mice. Interestingly, while the core components of sickness (anorexia and motor impairments) were comparable between *E.coli*-infected wild-type and PKR knockout mice, the behavioural components of sickness – including reduced burrowing, exploratory activity deficits, and social withdrawal – were only observed in PKR knockout mice (91).

Finally, PKR might also regulate apoptotic cell death during *E. coli* infection. *E. coli* RNA was shown to activate PKR and induce apoptosis of cardiac cells, an effect that was blocked following pharmacological PKR inhibition (86) **(** **Table 1** **)**. Altogether, the findings from the studies discussed above suggest that PKR regulates cell death pathways such as pyroptosis and apoptosis during *E. coli* infection. However, the effect of PKR expression on bacterial burden remains unclear, as there are discrepancies between the current findings.

### Yersinia pseudotuberculosis

*Yersinia pseudotuberculosis* is a Gram-negative, extracellular bacterium that causes Far East scarlet-like fever in humans. PKR is suggested to play a role in regulating inflammation and macrophage apoptosis during *Y. pseudotuberculosis* infection. Shrethsa and colleagues observed that EIF2α is phosphorylated in *Y. pseudotuberculosis*-infected RAW264.7 macrophages, and that phosphorylated EIF2α opposed bacterial invasion (103). Furthermore, phosphorylation of EIF2α was required for *Yersinia*-induced NF-κB activation and TNFα expression in MEFs. These findings indicated that PKR or another EIF2α kinase is involved in antibacterial defense against *Y. pseudotuberculosis*. Indeed, expression of the *Yersinia* virulence factor YopJ, which is known to inhibit EIF2α signaling in response to various stress stimuli, was also shown to inhibit PKR signalling in MEFs (103). This finding suggests that PKR is the kinase responsible for phosphorylating EIF2α during *Y. pseudotuberculosis* infection. PKR might also play a role in *Yersinia*-induced apoptosis, since deletion of PKR in infected BMDMs substantially impaired macrophage apoptosis when compared to wild-type macrophages (87) **(** **Table 1** **)**. The apoptotic response to *Y. pseudotuberculosis* was considerably reduced in BMDMs from TLR4-deficient mice, suggesting that PKR is required for TLR4-dependent apoptosis during *Yersinia* infection (103). The effect of PKR activity on the survival of *Y. pseudotuberculosis* has yet to be determined.

### Chlamydia trachomatis

*Chlamydia trachomatis* is a Gram-negative, obligate intracellular bacterium that is the causative agent of chlamydia. There is growing evidence to suggest that PKR is involved in the immune response to *C. trachomatis*. Shrestha and colleagues first reported that expression of a non-phosphorylatable mutant of EIF2α increased *C. trachomatis* invasion in MEFs, indicating that PKR or another EIF2α kinase assists in antibacterial defense against *Chlamydia* (103). They later reported that PKR knockout MEFs had increased *C. trachomatis* invasion levels compared to wild-type cells, with invasion levels similar to the levels observed in cells expressing the non-phosphorylatable EIF2α mutant (92) **(** **Table 1** **)**. These findings indicate that PKR plays a role in the antibacterial response to *C*. *trachomatis*. Indeed, a later study revealed that *C. trachomatis* infection triggers phosphorylation of PKR in human monocyte-derived dendritic cells and murine BMDMs, and this activation of PKR is required for *C. trachomatis*-induced IFNβ production (93) **(** **Table 1** **)**. This suggests that PKR plays a role in regulating inflammation during *Chlamydia* infection, but whether this PKR-dependent induction of IFNβ is ultimately conducive or detrimental to bacterial survival was not explored. However, findings from Qiu et al. indicate that the type I IFN-inducing role of PKR is conducive for *C. trachomatis* infection (110). *IFNAR* knockout mice were more resistant to *Chlamydia* infection compared to wild-type mice, as indicated by a smaller decrease in body weight, lower bacterial burden, and milder lung pathology in infected mice. The increased resistance to *C. trachomatis* infection in the knockout mice was attributed to higher numbers of bactericidal macrophages in the lung resulting from decreased macrophage apoptosis. Notably, these knockout mice had lower expression of PKR. Since PKR plays a proapoptotic role during viral infection, the authors speculate that type I IFN indirectly promote *C. trachomatis* infection by activating PKR, thus resulting in macrophage apoptosis and increased bacterial persistence (110). Additional experimentation is required to assess whether PKR plays a proapoptotic role during *C. trachomatis* infection. Altogether, PKR is activated by *Chlamydia* infection, where it then induces IFNβ production and limits bacterial invasion. Further investigation is necessary to determine whether PKR activity in response to *Chlamydia* infection is ultimately beneficial or harmful to the host.

### Legionella pneumophilia

*Legionella pneumophilia* is a Gram-negative, facultative intracellular bacterium that is the causative agent of Legionnaires’ disease. To examine the role of PKR during *L. pneumophilia* infection, Mallama and colleagues generated PKR knockdown cells using the human U937 macrophage-like cell-line (94). PKR deficiency impaired IL-6 secretion in response to *L. pneumophilia* infection **(** **Table 1** **)**, a critical cytokine in antibacterial defense against this bacterium. As such, Mallama et al. concluded that PKR expression is required for an optimal cytokine response to *L. pneumophilia* infection (94). However, PKR deficiency did not affect the intracellular burden of *L. pneumophilia* in macrophages **(** **Table 1** **)**. As such, although PKR appears to regulate inflammation during *L. pneumophilia* infection, the kinase may be dispensable for bacterial control.

## PKR in Mycobacterial Infections

### *Mycobacterium tuberculosis* Complex

*Mycobacterium* is a genus comprising over 190 species of bacteria. Mycobacteria possess an atypical outer membrane structure and organization comprised of mycolic acids, arabinogalactan, and numerous unique glycolipids (111). This unique cell wall contributes to the robustness of mycobacterial species and their natural tolerance to many antibiotics (112). As such, mycobacteria do not stain Gram-positive or Gram-negative, which contributes to their phylogenetic ambiguity. Instead, mycobacterial species are classified as acid-fast bacteria due to their ability to resist acid or ethanol-based decolorization procedures during staining. The most notorious species of mycobacteria relevant for human disease is *Mycobacterium tuberculosis*, a facultative intracellular bacterium that causes tuberculosis (TB) disease. Mycobacteria can be broadly classified into three major groups: the *Mycobacterium tuberculosis* complex, which comprises mycobacteria that cause TB disease (e.g. *M. tuberculosis* and *Mycobacterium bovis*); *Mycobacterium leprae*, which causes leprosy; and non-tuberculosis mycobacteria, which includes all other mycobacteria that do not cause TB or leprosy. There is growing evidence that PKR plays a role in the immune response to mycobacteria. For example, one study revealed that PKR phosphorylation is triggered in human monocytes infected with bacillus Calmette-Guérin (BCG), a live attenuated form of *M. bovis* used for TB vaccination (95). Pharmacological inhibition of PKR decreased mRNA and protein levels of crucial anti-BCG cytokines in infected monocytes, including TNFα, IL-6, and IL-10 **(** **Table 1** **)**. PKR inhibition also prevented the binding of NF-κB to DNA and impaired downstream activation of the MAPK ERK1/2 in treated monocytes. As such, the authors speculate that PKR induces anti-BCG cytokine production *via* downstream activation of ERK1/2 and NF-κB (95). Other studies have shown that *EIF2AK2* mRNA increases during infection with BCG and *M. tuberculosis* (113, 114). The findings that PKR expression and activation is triggered by mycobacterial infections suggest that PKR plays a role in the immune response to mycobacteria. However, the effect of PKR on mycobacterial burden was not examined in these studies.

Since PKR plays a pro-apoptotic role during viral infection, Wu and colleagues examined the effect of PKR deletion on macrophage apoptosis and bacterial burden during *M. tuberculosis* infection (115). While they initially reported that PKR deficiency in mice enhances macrophage apoptosis and decreases *M. tuberculosis* burden in the lungs (115), there was a discrepancy between the genetic backgrounds of the mutant and control mice used in the study (116). A follow-up study led by Mundhra and colleagues using mutant and control mice from the same genetic background revealed that PKR deficiency had no effect on apoptosis or *M. tuberculosis* burden (96, 116) **(** **Table 1** **)**. Therefore, PKR was concluded to be dispensable during *M. tuberculosis* infection. In contrast, Ranjbar and colleagues demonstrated that PKR expression and activation is triggered during *M. tuberculosis* infection in THP-1 monocytes, and PKR deletion in *M. tuberculosis*-infected macrophages increased the bacterial burden (97) **(** **Table 1** **)**. Although Ranjbar and colleagues observed an effect of PKR modulation on *M. tuberculosis* burden, the specific mechanism regulated by PKR to restrict *M. tuberculosis* growth was not investigated. As such, our group sought to examine the effect of PKR modulation on the intracellular survival of *M. tuberculosis* and characterize the specific mechanism(s) involved. Consistent with the findings from Ranjbar and colleagues, we showed that PKR expression and activation is triggered in *M. tuberculosis*-infected THP-1 and primary human macrophages, and deletion of PKR increases intracellular *M. tuberculosis* survival compared to control macrophages (98) **(** **Table 1** **)**. Strikingly, we also observed that genetic overexpression of PKR decreases the intracellular survival of *M. tuberculosis* by nearly 80% compared to control macrophages **(** **Table 1** **)**. Immunological profiling of infected macrophages overexpressing PKR showed increased production of IP-10 and reduced production of IL-6, two cytokines that are reported to activate and inhibit IFNγ-dependent autophagy, respectively (117, 118). Indeed, we determined that the ability of PKR overexpression to limit intracellular *M. tuberculosis* survival is due to the induction of selective autophagy (98) **(** **Table 1** **)**. Although our group did not elucidate the events downstream of PKR activation that led to induction of autophagy, the mechanism is likely through the phosphorylation of EIF2α and downstream induction of ATF4. However, MAPK p38 and JNK have been shown to be important for induction of autophagy during *M. tuberculosis* infection, and IL-6 inhibits MAPK phosphorylation to block autophagy in *M. tuberculosis*-infected macrophages (118). Importantly, PKR activates p38 and JNK (25–27), and macrophages overexpressing PKR had reduced production of IL-6 (98). As such, it is possible that autophagy induction by PKR is dependent on a mechanism involving MAPK, whether by a direct effect of PKR on MAPK activation or an indirect effect from decreased IL-6 production. We did not observe an effect of PKR modulation on apoptosis or overall cell death of *M. tuberculosis*-infected macrophages (98), consistent with the findings from Mundhra and colleagues (96). Taken together, the results from these studies indicate that PKR plays a critical role in the antibacterial response to mycobacteria. The ability of PKR to limit the intracellular survival of *M. tuberculosis* in macrophages appears to be through selective autophagy induction, rather than by regulating apoptosis.

It is worth noting that the overall effect of PKR during *M. tuberculosis* infection may be context dependent. Although our findings and the results from Ranjbar et al. indicate that PKR limits *M. tuberculosis* survival *in vitro* (97, 98), a recent report using *sst1*-susceptible mice suggests that PKR contributes to macrophage necrosis in TB granulomas (119). *sst1*-susceptible mice develop necrotic inflammatory lung lesions similar to human TB granulomas, whereas their congenic B6 counterparts do not (120). PKR phosphorylation was increased in TNFα-treated macrophages extracted from *sst1*-susceptible mice compared to macrophages extracted from B6 mice (119). PKR-mediated phosphorylation of EIF2α led to hyperinduction of ATF3 and integrated stress response (ISR)-target genes in these macrophages. Importantly, pharmacological inhibition of the ISR prevented the development of necrosis in lung granulomas of *M. tuberculosis*-infected *sst1*-susceptible mice and reduced the bacterial burden (119). This finding suggests that PKR contributes to necrosis of granulomas and subsequent lung pathology during TB disease. Further investigation of the effects of PKR modulation *in vivo* is required to assess whether PKR activity is ultimately beneficial or harmful to the host during *M. tuberculosis* infection.

### Non-Tuberculosis Mycobacteria

Non-tuberculosis mycobacteria (NTM) are mycobacteria that do not cause TB or leprosy. However, these mycobacteria can still cause illness in humans and animals, such as pulmonary disease resembling TB, lymphadenitis, and skin disease (121). NTM are mostly environmental bacteria and can be found in water and soil. Although most studies on PKR during mycobacterial infection used *M. tuberculosis* or BCG, there is limited evidence to indicate that PKR also plays a role during NTM infections. For instance, Madhvi et al. recently reported that *EIF2AK2* mRNA increases during infection with the non-pathogenic *Mycobacterium smegmatis* (114). In addition, there is evidence to loosely suggest that PKR plays a role in the immune response to *Mycobacterium ulcerans*, the causative agent of the tropical disease Buruli ulcer*. M. ulcerans* secretes an exotoxin virulence factor known as mycolactone, which triggers apoptosis of host cells (122). Cells that are exposed to mycolactone can persist for several days through the induction of autophagy before succumbing to apoptosis. However, chronic exposure to mycolactone causes cell death (123). Ogbechi and colleagues observed that MEFs with a deletion of both PERK and GCN2 (*Perk* ^-/-^ *Gcn2* ^-/-^) succumbed to apoptosis faster than wild-type cells, which was attributed to an inability of these cells to induce autophagy (123). Furthermore, mycolactone treatment increased protein levels of ATF4 and CHOP and triggered phosphorylation of PKR, PERK, GCN2, and EIF2α. Taken together, these findings suggest that mycolactone triggers phosphorylation of EIF2α kinases to activate the EIF2α-ATF4-CHOP pathway, which results in induction of autophagy during short-term exposure to the exotoxin, and induction of apoptosis during chronic exposure. Although the effect of PKR deletion on mycolactone-induced autophagy and apoptosis was not examined, residual *Atf4* expression was observed in *Perk* ^-/-^ *Gcn2* ^-/-^ MEFs (123). This suggests that PKR contributes to the phosphorylation of EIF2α and downstream induction of *Atf4* expression in response to mycolactone treatment. As such, it is possible that PKR plays a role in regulating autophagy and apoptosis during infection with *M. ulcerans*, although further experimentation using the live bacterium is required.

## Non-Canonical Activators of PKR

### Bacterial RNA

**Figure 2:**
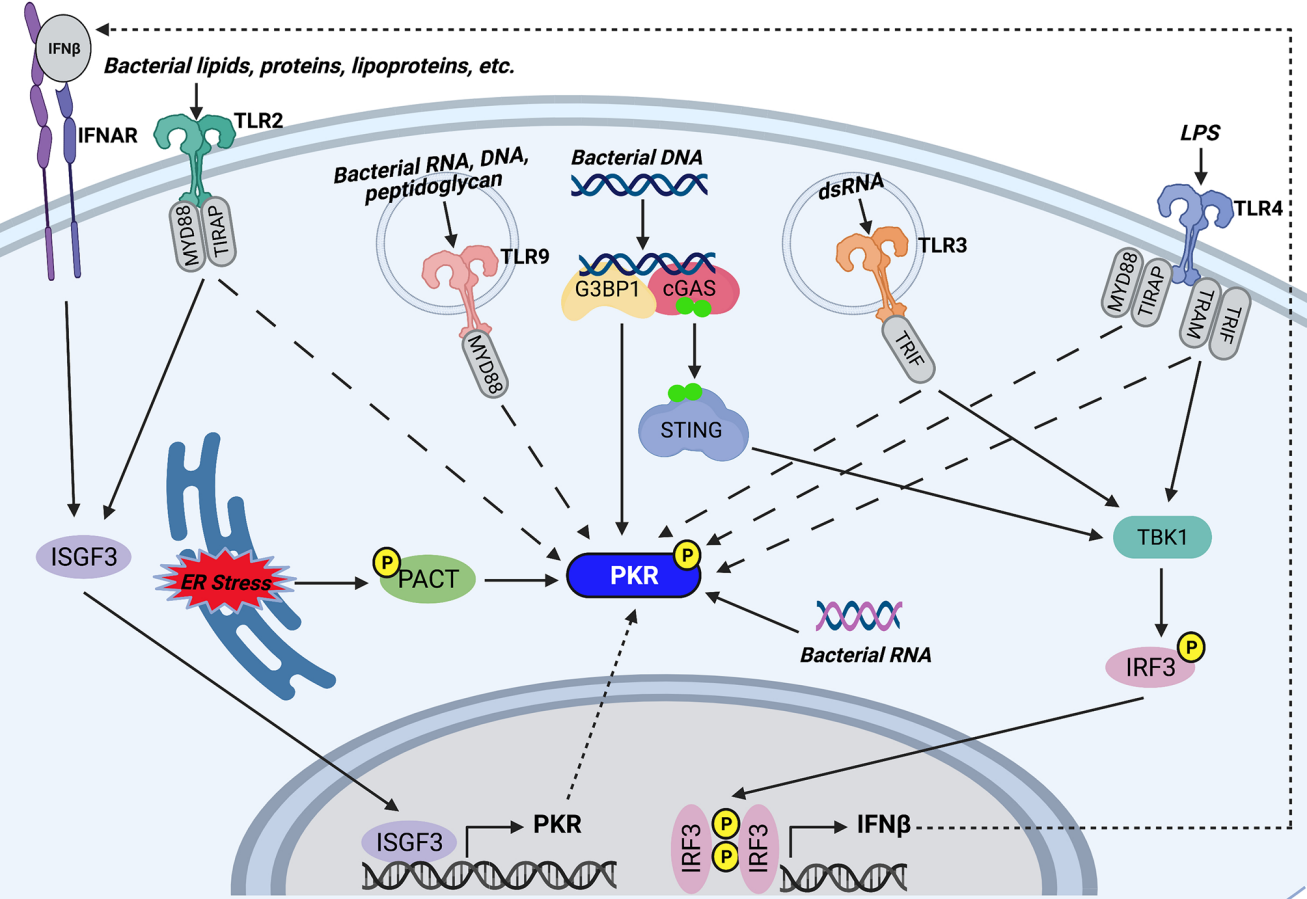
Potential mechanisms of PKR activation during bacterial infections. TLR2 and TLR4 on the host cell surface recognize bacterial lipids, proteins, and lipoproteins, or lipopolysaccharide (LPS), respectively. Endosomal TLR9 recognizes CpG motifs found in bacterial RNA, DNA, and peptidoglycan, whereas endosomal TLR3 is activated by bacterial dsRNA. TLR2, TLR4, TLR9, and TLR3 trigger PKR phosphorylation by unknown mechanisms. Furthermore, TLR3 and TLR4 activate TBK1 by their adaptor proteins (TRIF for TLR3 and TRAM and TRIF for TLR4) which goes on to induce phosphorylation and dimerization of IRF3. IRF3 translocates to the nucleus and induces transcription of IFNβ. IFNβ signals through IFNAR to trigger assembly and nuclear translocation of ISGF3, which regulates the PKR promoter to induce transcription of PKR. TLR2 signaling also triggers association of ISGF3 with the PKR promoter. As such, TLR3, TLR4, and TLR2 may induce PKR transcription by downstream activation of ISGF3. Cytosolic bacterial DNA is recognized by cGAS, which associates with G3BP1. G3BP1 co-localizes with PKR and may directly phosphorylate the kinase. Recognition of bacterial DNA triggers cGAS to synthesize the secondary messenger cyclic GMP-AMP. GMP-AMP triggers activation and dimerization of STING, which in turn activates the TBK1-IRF3-IFNβ-ISGF3 axis to induce PKR transcription. In addition, certain bacteria induce ER stress, which triggers phosphorylation of PACT, a cellular activator of PKR. Phosphorylation of PACT enhances its association with PKR and leads to PKR activation. Finally, cytosolic bacterial RNA can directly bind PKR and trigger its activation. Created with BioRender.com.

Although viral dsRNA is the canonical activator of PKR, recent studies have shown that PKR can also be activated by bacterial RNA. PKR was first suspected to be activated by bacteria when it was observed that purified PKR from *E. coli* cells is in a phosphorylated state and must be dephosphorylated to make the kinase responsive to RNA (124, 125). This suggested that endogenous bacterial products could trigger PKR activation. In 2012, Bleiblo and colleagues identified bacterial RNA as a ligand recognized by PKR (86). It was observed that total RNA extracted from *E. coli* and *S. aureus* potently activated PKR in cardiac cells in a dose-dependent manner, whereas human RNA did not. *In vitro* PKR binding assays showed that the bacterial RNA directly bound the purified PKR, suggesting that bacterial RNA possesses structural features that can directly activate PKR (86) **(** **Figure 2** **)**. A later study from the same group demonstrated that removal of the base-paired secondary structures of the bacterial RNA by RNase digestion hinders the activation of PKR, indicating that the double-stranded structures of bacterial RNA are required to fully activate PKR (126). More recently, Hull and colleagues investigated the specific features of bacterial RNA that can activate PKR (127, 128). The *Bacillus subtilis trp* 5’-UTR was identified as an activator of PKR. The *trp* 5’UTR has multiple structural RNA elements representative of many bacterial mRNAs, including a terminator, 5’-stem-loop, and Shine-Dalgarno hairpin. These elements were shown to potently activate PKR. In a follow-up study by Hull and colleagues, three more functional bacterial RNAs were tested for their ability to activate PKR: the Vc2 riboswitch from *Vibrio cholerae*, the *glmS* riboswitch-ribozyme from *B. anthracis*, and the twister ribozyme from *Clostridia bolteae* (128). Most constructs derived from these RNAs were able to activate PKR, provided they were long enough to form sufficient RNA structure. These findings demonstrate that PKR can be activated by numerous RNA elements from a wide range of bacteria, including both Gram-positive and Gram-negative bacteria.

### DNA

The finding that PKR is required for AIM2 inflammasome activation in response to DNA transfection suggested that PKR can be activated by double-stranded DNA (dsDNA) (62), but PKR does not bind dsDNA or DNA/RNA hybrid strands (129). Nevertheless, DNA may activate PKR indirectly. Indeed, one study showed that transfection of HeLa cells with exogenous DNA led to phosphorylation of both PKR and EIF2α (130). This effect was dependent on recognition of dsDNA by the cytosolic DNA sensor cGAS. It was also observed that PKR and G3BP1, an RNA/DNA and RNA/RNA helicase, co-localize with cGAS following DNA transfection and are both required for cGAS sensing of intracellular vaccinia virus DNA. Interestingly, G3BP1 was required for PKR phosphorylation. As such, the authors of this study suggest that after interacting with DNA-bound cGAS, G3BP1 activates PKR (130) **(** **Figure 2** **)**. It is note-worthy that the cGAS/STING/TBK-1 axis stimulated by cytosolic dsDNA leads to activation of IRF3, which induces transcription of IFNβ (131). IFNβ triggers assembly and nuclear translocation of the transcription factor interferon-stimulated gene factor 3 (ISGF3), which regulates the PKR promoter at the ISRE (132). Therefore, it is also possible that the DNA-sensing pathway indirectly induces PKR transcription by triggering IFNβ production and downstream ISGF3 activation **(** **Figure 2** **)**. Altogether, we speculate that the cytosolic DNA sensing pathway is a potential mechanism that triggers PKR expression and phosphorylation during bacterial infection.

### Toll-Like Receptor Signalling

Pathogens that are unable to perforate the phagosome, such as BCG and LLO-deficient *L. monocytogenes*, can trigger PKR phosphorylation and mRNA expression (89, 95). This suggests that PKR can be activated/induced in the absence of cytosolic nucleic acids. Indeed, there is accumulating evidence to support that PKR is activated by TLR signaling. PKR is reported to be activated in response to TLR4 and TLR2, which are cell surface TLRs that mainly recognize microbial membrane components such as lipids, lipoproteins, and proteins. In 2001, Horng and colleagues observed that PKR is phosphorylated following treatment with LPS, a TLR4 agonist (133). Since then, there have been numerous reports of PKR activation occurring in a TLR4-dependent manner (87, 93, 134–138). TLR4 signaling has also been shown to increase *EIF2AK2* mRNA and protein levels (135). As such, we speculate that bacteria can trigger downstream PKR phosphorylation and expression by activating TLR4 signaling **(** **Figure 2** **)**. Indeed, a link between TLR4 signaling and PKR activity has been reported during infection with bacteria such as *S.* Typhimurium, *Y. pseudotuberculosis*, *B. anthracis*, and *C. trachomatis* (87, 93). Importantly, both heat-treated and gamma-irradiated attenuated *C. trachomatis* can activate PKR to the same extent as live bacteria, indicating that intracellular bacterial replication or secretion of heat-labile bacterial products are not responsible for PKR activation (93). In contrast, inhibition of TLR4 prevented PKR activation (93). Altogether, these findings provide evidence that heat stable LPS is the likely bacterial product responsible for PKR activation during *C. trachomatis* infection. Indeed, heat-treating LPS did not impair its ability to activate PKR (93). Interestingly, PKR appears to be activated by different adaptor proteins depending on the specific TLR4 agonist (93, 133). *C. trachomatis* infection is unable to activate PKR in the presence of a MyD88 inhibitor but is unaffected by a TRIF inhibitor, whereas LPS is unable to activate PKR in the presence of a TRIF inhibitor but is unaffected by a MyD88 inhibitor (93). TLR2 signaling can also trigger PKR phosphorylation **(** **Figure 2** **)**. Pam3CSK4, a TLR2 agonist, triggers PKR phosphorylation (134) in addition to increasing *PKR* mRNA and protein levels (135). Furthermore, PKR is activated in a TLR2-dependent manner following parasitic infection with *Leishmania amazonensis* (139).

PKR phosphorylation can also be triggered downstream of TLR3 and TLR9, which are endosomal TLRs that mainly recognize nucleic acids **(** **Figure 2** **)**. Indeed, a kinase-inactive PKR mutant inhibited poly(I:C)-induced TLR3-mediated activation of NF-κB, suggesting that PKR is a downstream component of TLR3 signaling (140). A later study confirmed that PKR is activated by TLR3 signaling, since poly(I:C)-induced phosphorylation of PKR in human neuroblastoma cells was impaired in TLR3-deficient cells (141). There is limited evidence to suggest that PKR is activated in response to TLR9 signaling. PKR was shown to be phosphorylated by CpG motifs present in bacterial DNA, dsRNA, and peptidoglycans (133). CpG engages the TLR9 receptor, indicating that PKR is activated downstream of TLR9 **(** **Figure 2** **)**. As such, TLR9 signaling could be yet another mechanism through which PKR indirectly senses bacterial DNA without a requirement for phagosome perforation. Overall, PKR appears to be a downstream component shared by at least four TLRs.

The specific mechanism of PKR activation downstream of TLR signaling remains unclear. However, Perkins and colleagues observed that TLR4 and TLR3 agonists trigger the phosphorylation of both PKR and IRF3 (138). IRF3 induces transcription of IFNβ, which triggers assembly and nuclear translocation of ISGF3. As mentioned in the previous section, ISGF3 regulates the PKR promoter (132); therefore, it is possible that TLR4 and TLR3 signaling induces PKR by triggering IFNβ production and downstream ISGF3 activation **(** **Figure 2** **)**. Furthermore, chromatin immunoprecipitation assays revealed that *L. amazonensis* infection, which activates PKR through TLR2 signaling, triggered binding of ISGF3 elements to the PKR promoter, an event that did not occur in TLR2 knockout macrophages (139). Therefore, ISGF3 may be a common link between TLR2-, TLR4-, and TLR3-dependent induction of PKR **(** **Figure 2** **)**.

### ER Stress

There are numerous reports that PKR is activated in response to ER stress (142–144). Indeed, PKR has been shown to play a significant role in sustained ER stress-induced apoptosis (54). ER stress leads to downstream PKR activation by triggering the phosphorylation of PACT (54), a cellular activator of PKR that activates the kinase in the absence of dsRNA (55). Phosphorylation of PACT increases its association with PKR and leads to PKR activation. Certain bacteria, including *Pseudomonas aeruginosa*, *Helicobacter pylori*, and *Coxiella burnetti*, are reported to trigger ER stress in infected host cells (145–148). Although the phosphorylation state of PKR was not examined in these studies, PERK and EIF2α were shown to be phosphorylated in response to infection with these bacteria. It is possible that PKR is also activated in response to these bacteria and contributes to the phosphorylation of EIF2α. Indeed, one of the groups suggested that future work should assess whether PKR plays a role in the ER stress response during *C. burnetti* infection (146). As such, we speculate that infection with certain bacterial pathogens induces ER stress, which leads to PKR activation *via* PACT **(** **Figure 2** **)**.

## Pharmacological Modulation of PKR

### PKR Activators

Due to the emergence of antibiotic-resistant bacteria such as *M. tuberculosis* and *S. aureus*, the development of alternative therapies for certain bacterial infections is urgently required. Host-directed therapy (HDT) is a promising option, since this strategy aims to boost the host immune response to a particular bacterium rather than target the bacterium itself, thus circumventing the development of antibiotic resistance. Since there is growing evidence that PKR plays a role in the host immune response to bacterial pathogens, pharmacological modulation of PKR could be a promising strategy for HDT against various bacterial infections.

There are multiple pharmacological activators of PKR in various stages of development **(** **Table 2** **)**. Bozepinib is a small antitumor agent that both upregulates and activates PKR (149). Bozepinib has shown promise in pre-clinical studies since it induces apoptosis in breast and colon cancer cells (149, 150). Although the specific effects of bozepinib have not been assessed *in vivo*, it has been observed that bozepinib treatment does not cause acute toxicity in mice (150). The mechanism through which bozepinib induces and activates PKR remains unknown. Nitazoxanide (NTZ) is another drug that triggers PKR phosphorylation (151, 152). NTZ is an FDA-approved broad-spectrum antiparasitic drug. The typical use of NTZ is the treatment of cryptosporidiosis infection, however clinical trials have demonstrated efficacy and safety of NTZ in treating viral infections such as influenza and hepatitis C (188, 189). NTZ has been shown to deplete intracellular calcium stores, thereby raising levels of cytosolic calcium. This calcium mobilisation disrupts ER/Golgi glycoprotein trafficking and induces ER stress, thus triggering PKR phosphorylation (152). Several *in vivo* studies conducted in animal models have investigated the effects of NTZ in disease contexts such as viral infections, protozoan infections, cancer, Parkinson’s disease, neuroinflammation, and bacterial infections **(** **Table 2** **)** (153–166). Indeed, *in vivo* studies have shown that NTZ is effective in treating bacterial pathogens such as *C. difficile*, *E. coli*, *M. leprae*, and *M. tuberculosis* (153–156, 159). Interestingly, NTZ is reported to exert significant bactericidal activity directly against both replicating and non-replicating *M. tuberculosis* (190), and was recently evaluated for treatment of TB in a phase II clinical trial (191). In addition, Ranjbar and colleagues recently reported that NTZ treatment enhanced *M. tuberculosis*-induced *EIF2AK2* mRNA expression in THP-1 cells, and NTZ treatment reduced *M. tuberculosis* burden in THP-1 and human peripheral blood mononuclear cells (97) **(** **Table 1** **)**. As such, NTZ is a PKR activator that holds promise for being repurposed as a host-directed antibacterial drug. The synthetic compound BEPP [1*H*-benzimidazole1-ethanol,2,3-dihydro-2-imino-*a*-(phenoxymethyl)-3-(phenylmethyl)-,monohydrochloride] is another PKR activator that increases PKR and EIF2α phosphorylation in a dose-dependent manner in MEFs (167). Interestingly, BEPP was shown to induce PKR-dependent apoptosis and effectively inhibited vaccinia virus replication in MEFs (167). However, the effects of BEPP have not been studied *in vivo*, and the mechanism of action of BEPP on PKR activity is unknown. Lastly, 3-(2,3-dihydrobenzo[b][1,4]dioxin-6-yl)-5,7-dihydroxy-4H-chromen-4-one (DHBDC) is a dual activator of PKR and PERK (168). DHBDC was shown to induce the phosphorylation of EIF2α, which was blocked by siRNAs targeting PKR and PERK. The mechanism by which DHBDC activates PKR remains unknown, and the effects of this compound have not been assessed *in vivo*. Both BEPP and DHBDC are commercially available for research use.

**Table 2:** Pharmacological modulators of PKR.

	Compound	Method of PKR modulation	Stage of development	Animal model	Disease context	Citation
**Activators**	Bozepinib	Unknown	Pre-clinical	N/A	N/A	(149, 150)
	Nitazoxanide	Depletes intracellular Ca^2+^ stores, resulting in ER stress and PKR phosphorylation	FDA approved	Rats, mice, hamsters	Microbial infections cancer, inflammation, neuropathic pain, Parkinson’s disease	(151–166)
	BEPP	Unknown	Pre-clinical	N/A	N/A	(167)
	DHBDC	Unknown	Pre-clinical	N/A	N/A	(168)
**Inhibitors**	C16	Competitive inhibitor of ATP	Pre-clinical	Rats, mice	Neurodegeneration, hypertension, cancer, diabetes, rheumatoid arthritis, inflammation	(169–184)
	2-Aminopurine	Competitive inhibitor of ATP	Pre-clinical	Mice	Inflammation, diabetes	(170, 185–187)

N.A, not applicable.

### PKR Inhibitors

There are currently two pharmacological inhibitors of PKR being investigated in pre-clinical studies: imidazole-oxindole C16 and 2-aminopurine (2-AP) **(** **Table 2** **)**. Both C16 and 2-AP compete for ATP at the ATP binding site of PKR, thus inhibiting PKR autophosphorylation and kinase activity (169, 185). The most widely-used PKR inhibitor is C16, also known as PKRi or Imoxin (169). C16 has been shown to inhibit PKR phosphorylation *in vitro* and in the mouse model (169–171). The effects of this compound have been examined in numerous *in vitro* studies (5). Furthermore, several *in vivo* studies using mice and rats have examined the effect of C16 in disease contexts such as inflammation, neurodegeneration, obesity, hypertension, cancer, and diabetes (170–184) **(** **Table 2** **)**. Importantly, numerous groups have shown that C16 is protective of LPS-induced pathogenesis in mice, including acute lung injury, bone destruction, skeletal muscle atrophy, and acute kidney injury (177–181). Since LPS is a major cell wall component of Gram-negative bacteria, these findings suggest that C16 could protect against excessive inflammation and tissue damage during bacterial infections. However, it is also possible that the anti-inflammatory effect of C16 treatment could exacerbate disease progression in the context of live bacterial infections due to the dampening of the immune response. 2-AP is a less potent and less specific PKR inhibitor (185). In mouse models, 2-AP has been shown to prevent sepsis induced by cecal ligation puncture or endotoxin challenge (186, 187). Furthermore, 2-AP treatment reduces adipose tissue inflammation and improves insulin sensitivity in insulin-resistant obese mice (170). Since 2-AP has anti-inflammatory effects, this drug holds promise in treating bacterial infections where excessive inflammation is conducive for the pathogen. Both C16 and 2-AP are commercially available for research use.

### Potential Challenges

There are numerous reports demonstrating that PKR plays an important role during bacterial infections **(** **Table 1** **)**, which suggests that pharmacological modulation of PKR could be a promising strategy for host-directed therapy. However, the vast majority of these studies were conducted *in vitro*. Out of the 14 studies listed in **Table 1**, only 4 studies were conducted *in vivo* (62, 90, 91, 96). Notably, these studies only examined the bacterial burden in organs of infected mice and did not assess the overall survival of the animals. Indeed, while there exist numerous pharmacological activators and inhibitors of PKR, only NTZ has been tested *in vivo* in the context of bacterial infections **(** **Table 2** **)**. As such, extensive *in vivo* experimentation must be conducted before promoting the use of pharmacological PKR modulation as a therapeutic intervention against bacterial pathogens. It will be critical to demonstrate that pharmacological modulation of PKR has the ability to improve host survival during bacterial infection in animal models. Furthermore, *in vivo* studies must be conducted to identify any limitations of using pharmacological modulators of PKR.

One potential challenge of inhibiting PKR is that interventions that dampen the inflammatory response can sometimes enhance the susceptibility of the host to lethal bacterial infection. For example, C3H/HeJ mice, which have a defective LPS response, are highly susceptible to *E*. *coli* and *S*. Typhimurium infection (192). Since PKR expression is demonstrated to be important for inflammasome activation and induction of pro-inflammatory cytokines in response to bacterial infections **(** **Table 1** **)**, it is possible that pharmacological inhibition of PKR would ultimately be disadvantageous for the host. Another concern is the fact that PKR activity affects many different signaling pathways. This means that inhibiting or activating PKR-dependent pathways involved in the immune response to bacterial pathogens may also disrupt other PKR-dependent pathways that are essential for other functions. Indeed, dysregulation of PKR has been linked to numerous diseases, including neurodegeneration, cancer, and metabolic disorders (4–6). Furthermore, PKR regulates different immune functions depending on the context of the bacterial infection **(** **Table 1** **)**, which suggests that the kinase can play either a pro- or anti-host role during bacterial infection contingent on the specific bacterium involved. As such, it is possible that clinicians would be unable to administer pharmacological PKR modulators to patients until the specific pathogen was identified, thus delaying the initiation of host-directed therapy until it is potentially too late. Altogether, these potential challenges highlight the necessity of evaluating the effects of PKR modulators *in vivo*.

## Concluding Remarks

There is growing evidence demonstrating that PKR plays key roles during infection with various bacterial pathogens **(** **Table 1** **)**. Indeed, current literature clearly demonstrates a role for PKR in regulating selective autophagy, cell death, and inflammation during bacterial infections. In response to both Gram-positive and Gram-negative bacterial infections, PKR expression has been shown to be important for inflammasome activation, pyroptosis, and apoptosis. In contrast, PKR is not observed to regulate cell death pathways during mycobacterial infection, but is instead reported to induce selective autophagy. PKR has also been shown to regulate cytokine production in response to mycobacteria, Gram-positive, and Gram-negative bacteria. We speculate that the varying functions of PKR during bacterial infections is due to the specific bacterium involved, since bacterial pathogens have methods of manipulating host immune responses to their advantage. For instance, PKR expression is required for macrophage apoptosis during *B. anthracis* infection (87), but PKR modulation does not impact apoptosis during *M. tuberculosis* infection (98, 116). This is likely explained by the fact that a major virulence mechanism of *B. anthracis* is to induce rapid cell death of host cells (193, 194), whereas *M. tuberculosis* inhibits macrophage apoptosis to allow it to persist undetected within the phagocyte (195). Most studies thus far have focused on the role of PKR in cell death during bacterial infection. Although it is important to investigate the function of PKR during cell death, we contend that this should not be the sole focus, since PKR has recently been shown to induce selective autophagy during bacterial and parasitic infection (77, 98). As such, it will be important for future studies to investigate the function of PKR in selective autophagy of intracellular bacteria such as *L. monocytogenes* and *S*. *enterica*, since autophagy is reported to play a role in antibacterial defense against these pathogens (196–198).

Although PKR has critical functions in bacterial defense, whether the kinase is ultimately protective or detrimental to the host remains to be clarified. For instance, deletion of PKR led to decreased bacterial burden in organs of *E. coli*-infected mice (62) but resulted in increased bacterial burden in *M. tuberculosis*-infected macrophages (97, 98). Whether PKR plays a pro- or anti-host role during bacterial infection is likely dependent on the specific bacterium involved, and whether the bacterium establishes acute *versus* chronic infection. For example, *S. enterica* causes acute infection, and a strong inflammatory response in the early stage of infection is generally considered to assist with bacterial clearance (199, 200). In contrast, *M. tuberculosis* establishes chronic infection, therefore excessive and prolonged inflammation during infection with this bacterium can be harmful to the host (201). As such, we speculate that the function of PKR in inflammasome activation would be beneficial to the host during *S. enterica* infection, but harmful to the host during *M. tuberculosis* infection. Similarly, PKR is established to induce IFNβ production, which can be a pro- or anti-host function depending on the bacterial context. Indeed, IFNβ production is detrimental to the host during *L. monocytogenes* infection due to its role in inducing macrophage apoptosis (202), but protective for the host during *L. pneumophilia* infection since it promotes itaconic acid production (203).

Unfortunately, most of the existing studies of PKR in the context of bacterial infections examined either the effect of PKR on a particular host signaling pathway, or the effect of PKR expression on bacterial burden, but rarely were both examined within the same study **(** **Table 1** **)**. Furthermore, only a limited number of studies have examined the effect of PKR modulation on bacterial burden, with only a select few that included *in vivo* experiments **(** **Table 1** **)**. *In vivo* studies will be critical in determining whether PKR is ultimately protective or detrimental to the host during infection with a given bacterium. Importantly, *in vivo* experimentation of PKR is feasible given that pharmacological approaches have been vetted in other disease models **(** **Table 2** **)** and both genetic overexpression and deletion of PKR is well-tolerated in mice (204, 205). Both inhibition and activation of PKR by pharmacological compounds **(** **Table 2** **)** should be actively pursued given that modulation in either direction could be specifically harnessed for treatment of specific bacterial diseases. As such, future studies should examine the effects of pharmacological modulation of PKR on bacterial burden, morbidity, and mortality of bacteria-infected mice to assess the suitability and feasibility of targeting PKR as a novel treatment strategy. At the same time, it will also be important to determine the specific mechanism regulated by PKR during bacterial infection, since it will link the specific functional pathway to whether PKR is ultimately protective or detrimental to the host.

There also exists a knowledge gap on the specific downstream effectors mediated by PKR in response to various bacteria. For example, although our group observed that PKR induces autophagic degradation of *M. tuberculosis*, the downstream pathway activated by PKR in this context was not elucidated (98). PKR is reported to mediate the activation of numerous downstream effectors, including MAPK, ATF2, NF-κB, IPS-1, IRF3, ATF4, and CHOP **(** **Figure 1** **)**. As such, there are many potential mechanisms controlled by PKR to regulate cellular processes such as autophagy, inflammation, and cell death in response to bacterial infections. Future studies should elucidate the specific downstream components regulated by PKR during the immune response to bacterial pathogens.

The upstream signaling events that trigger PKR activation during bacterial infection also need to be determined. Although the canonical activator of PKR is viral dsRNA, the ability of certain phagosome-restricted bacteria to activate PKR suggests the existence of alternative activating mechanisms (89, 95). In this review, we discussed non-canonical activators of PKR – including TLR signaling, ER stress, and bacterial nucleic acids – and speculated on potential mechanisms that trigger PKR activation during bacterial infection **(** **Figure 2** **)**. However, although exogenous treatment of cells with TLR agonists, bacterial RNA, or chemical inducers of ER stress can activate PKR, there is a lack of evidence to show that live bacteria trigger PKR activation through these specific mechanisms. Future studies examining PKR activity during bacterial infection should strive to characterize the mechanism responsible for activating PKR in this context.

In summary, PKR undoubtedly plays key roles during bacterial infections, as multiple studies have shown that PKR regulates critical immune cell functions such as inflammation, apoptosis, pyroptosis, and autophagy. Increasing our knowledge on the role of PKR during bacterial infections is important since it could lead to the development of host-directed therapies for antibiotic-resistant bacteria. Regardless of whether PKR activity is ultimately beneficial or detrimental to the host, modulating its expression or activity holds promise as a novel treatment strategy for bacterial infections.

## Summary

- PKR regulates cell death in both Gram-positive and Gram-negative bacterial infections by inducing apoptosis and activating the inflammasome to trigger pyroptosis.
- PKR regulates the production of multiple cytokines with key roles in antibacterial defense, including Type I IFNs, IL-1β, IL-6, IL-10, IL-18, and TNFα.
- PKR induces selective autophagy during *M. tuberculosis* infection.
- Conflicting reports exist on whether PKR is protective or detrimental to the host. This is likely due to (i) different bacterial pathogens involved, (ii) the specific infection model used for each study, and (iii) the specific mechanistic pathway at play (i.e. cell death *vs*. autophagy).
- The mechanisms of PKR activation during bacterial infection remain to be elucidated, but may involve bacterial nucleic acids, TLR signaling, or ER stress.
- Pharmacological modulation of PKR holds promise as an alternative treatment strategy for bacterial infections, but extensive *in vivo* studies must be conducted to assess the effects of PKR modulation on host survival and identify potential off-target effects.

## Author Contributions

RS and JS wrote and edited the manuscript. All authors contributed to the article and approved the submitted version.

## Funding

This work was supported by the Canadian Institutes of Health Research (CIHR) PJT-162424, the National Sanitarium Association Scholar’s Program, and the Natural Sciences and Engineering Research Council of Canada (NSERC) RGPIN-2020-04032 to JS.

## Conflict of Interest

The authors declare that the research was conducted in the absence of any commercial or financial relationships that could be construed as a potential conflict of interest.
